# ModE-RA: a global monthly paleo-reanalysis of the modern era 1421 to 2008

**DOI:** 10.1038/s41597-023-02733-8

**Published:** 2024-01-05

**Authors:** Veronika Valler, Jörg Franke, Yuri Brugnara, Eric Samakinwa, Ralf Hand, Elin Lundstad, Angela-Maria Burgdorf, Laura Lipfert, Andrew Ronald Friedman, Stefan Brönnimann

**Affiliations:** 1 Oeschger Centre for Climate Change Research, University of Bern, Bern, Switzerland; 2 Institute of Geography, University of Bern, Bern, Switzerland

**Keywords:** Palaeoclimate, Atmospheric science

## Abstract

The Modern Era Reanalysis (ModE-RA) is a global monthly paleo-reanalysis covering the period between 1421 and 2008. To reconstruct past climate fields an offline data assimilation approach is used, blending together information from an ensemble of transient atmospheric model simulations and observations. In the early period, ModE-RA utilizes natural proxies and documentary data, while from the 17^th^ century onward instrumental measurements are also assimilated. The impact of each observation on the reconstruction is stored in the observation feedback archive, which provides additional information on the input data such as preprocessing steps and the regression-based forward models. The monthly resolved reconstructions include estimates of the most important climate fields. Furthermore, we provide a reconstruction, ModE-RAclim, which together with ModE-RA and the model simulations allows to disentangle the role of observations and model forcings. ModE-RA is best suited to study intra-annual to multi-decadal climate variability and to analyze the causes and mechanisms of past extreme climate events.

## Background & Summary

Analyzing longer time periods than what is covered by modern instruments helps to gain further insights into the climate system, its variability and understanding of historical climate events. However, to study the period before the availability of state-of-the-art instrumental measurements, e.g., to analyze the intra-annual dynamics of past climate changes, datasets with a high temporal resolution are required. Thanks to various initiatives, several new climate reconstructions of the Common Era (the past 2000 years)^1–5^, most of them with annual resolution, have become available along with data compilations^6,7^, and model simulations. For the last 600 years, there exist enough high-resolution data – natural proxies (e.g., tree rings, coral, ice cores), documentary evidence, and since 1658 early instrumental measurements – to attempt to generate a monthly global climate reconstruction.

To access climate variations in the past we can use modeling results and observations, but neither of the two sources can fully capture past climate variability. On the one hand, model simulations can only give a possible range of past climate states but the specific realization of random, internal variability that occurred in the real world cannot be reproduced. On the other hand, observational data become sparser in space the earlier the studied period is, in line with decreasing temporal resolution. Many recent global climate reconstructions use data assimilation to provide a best estimate (so-called analysis) of past climate fields by combining model simulations and observations^1–3,5,8^. In this study, we employ an ensemble-based Kalman filter based data assimilation approach^9^ and use the newest data compilations with a new ensemble of atmospheric general circulation model simulations^10^ to derive monthly global atmospheric paleo-reanalyses from 1421 to 2008.

**Fig. 1:**
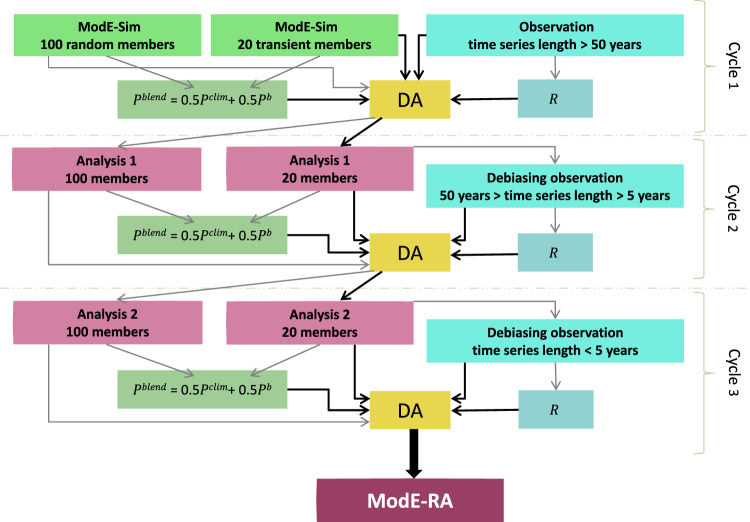
Schematic overview of the data assimilation steps involved to generate the ModE-RA paleo-reanalysis. P is the model/analysis-error covariance matrix estimated from the ensemble and R is the observation-error covariance.

Previous monthly climate reconstructions generated by our group based on a similar data assimilation technique date back to 1601^2,5^. Compared to these reconstructions, the amount of assimilated input data in this study largely exceeds the data used in previous products, we improved our data treatment strategy, and added a new method to assimilate short time series. Between 1421 and 1657 the observational network is exclusively based on proxy records and documentary evidence. The first long instrumental measurement series become available in Europe starting in 1658. With time the observational network becomes more and more dense, reaching a peak in the 1880s and 1890s, because after 1890 we do not add any new series to the network and many of these stations stopped measuring sometime in the 20^th^ century. The enlarged input database enables us to produce more robust reconstructions, for example over the winter seasons in the 17^th^ and 18^th^ centuries in the Northern Hemisphere due to the incorporation of documentary evidence and early instrumental data. The assimilated observations (temperature-sensitive and/or precipitation-sensitive proxy records, documentary evidences and early instrumental measurements over land and marine areas) have indirect or direct information about past temperature, pressure, precipitation, and wind. In addition to these variables, we reconstruct other climate fields of interest such as geopotential height and wind components on several vertical levels. Moreover, we provide a valuable observation feedback archive to be able to trace back the effect of the observations and at the same time to publish the input data. In order to quantify the effect of forced variability in the simulation on the reanalysis, we performed two reconstructions: one using an ensemble of transient model simulations as prior (ModE-RA), and one sensitivity experiment with random stationary priors (ModE-RAclim) (Table 1). A schematic overview of the implemented data assimilation system of ModE-RA is shown on Fig. 1.

**Table 1:** Summary of main features of the assimilation products and the model simulation.

	ModE-RA	ModE-RAclim	ModE-Sim
Members	20	100	20*
Time dependent forcings	yes	no	yes
Assimilation	yes	yes	no
Data links	ModE-RA	ModE-RAclim	ModE-Sim Set 1420-3 ModE-Sim Set 1850-1

*ModE-Sim has additional members. Here we refer to the 20 members used to generate ModE-RA.

ModE-RA is systematically evaluated by comparisons with independent time series, gridded instrumental observations, and other climate reconstructions. Most paleo-reanalyses are based on non-transient priors from climate simulations. Their low-frequency variability is derived from the assimilated empirical data. In contrast, the centennial-scale variability in ModE-RA originates from the model response to forcings; therefore this dataset is best suited to study intra-annual to multi-decadal variability. Observations were transformed to anomalies from 71-year running means to reduce trends due to the changing input data network through time and because centennial-scale climate variability is not consistently preserved in many of the assimilated proxy records, for instance due to an inconsistent age trends removal in tree-ring width^11^. ModE-RA shows good agreement with 20^th^ century gridded monthly products, for temperature (globally), sea-level pressure (especially in the Northern Hemisphere) and precipitation (in the regions where precipitation observations were assimilated). Comparing ModE-RA with gridded annual temperature reconstructions, we find the highest correlations with the summer mean (JJA) of ModE-RA. Evaluation against independent documentary evidence shows very similar values to the calibration results indicating a good performance of ModE-RA.

The overall goal of ModE-RA is to provide a multi-variate dataset that combines all available direct and indirect climate observations, with reconstructions of external forcings and the physics represented in climate models. In future studies, ModE-RA can be used to assess climate variability, historical climate events like volcanic eruptions and to examine past extreme events, such as several month long droughts, which may have no counterpart in the modern instrumental period.

## Methods

### Offline data assimilation

Data assimilation (DA) has been applied in multiple paleoclimatogical studies^1–3,12^, making it possible to use sparse data with a continuously-varying observational network and, at the same time, to produce realistic climate fields in accordance with the model physics. Among the various DA techniques, ensemble-based Kalman filter methods have particularly been used in paleoclimate reconstructions^1,2,13,14^. Most of the reconstructions of past climate are built on already-existing model simulations and do not propagate the analysis forward in time. This technique is known as offline DA. It has been previously argued that in the case of paleoclimate reconstruction, the predictability of the system is shorter than the temporal resolution of the observations, and no benefit was found from propagating the analysis forward if the focus is on land regions^13,15^. A recent study^16^ showed that online DA can outperform offline DA when it is coupled with an ocean model. However, applying an offline DA method is computationally much cheaper than the traditional online techniques, and it allows for easy testing. Thus, we keep working with the offline approach.

Various ensemble-based Kalman filter approaches have been developed over the past few decades^17–20^. We use the offline variant of the ensemble square root filter (EnSRF) DA approach^9^ to generate a 600-year-long global monthly atmospheric paleo-reanalysis (ModE-RA). Here, we briefly describe the update part of the EnSRF, in which the observation information is optimally combined with the model simulations. The EnSRF, like other ensemble-based approaches, uses the ensemble statistics to specify the Kalman filter equations. The update step of the EnSRF is divided into an update of the ensemble mean ($\bar{x}$) and an update of the deviations from the ensemble mean (${x}_{i}^{{\prime} }={x}_{i}-\bar{x}$):

1 $${\bar{x}}^{a}={\bar{x}}^{b}+K\left(y-H{\bar{x}}^{b}\right)$$

2 $${x}^{{\prime} a}={x}^{{\prime} b}-\widetilde{K}\left(H{x}^{{\prime} b}\right)$$

The background state vector (*x*^*b*^) represents the state of the atmosphere before the assimilation of empirical data. In the case of offline DA, *x*^*b*^ is obtained from existing model simulations. *x*^*a*^ is the analysis, the updated state where observation and model information have been merged. *H* is the forward operator, which maps the model values to the observed data. *K* and $\widetilde{K}$ represent the Kalman gain matrix and the reduced Kalman gain matrix, which are calculated as:

3 $${\rm{K}}={{\rm{P}}}^{{\rm{b}}}{H}^{{\rm{T}}}{(H{{\rm{P}}}^{{\rm{b}}}{H}^{{\rm{T}}}+{\rm{R}})}^{-1}$$

4 $$\widetilde{{\rm{K}}}={{\rm{P}}}^{{\rm{b}}}{H}^{{\rm{T}}}{({(\sqrt{H{{\rm{P}}}^{{\rm{b}}}{H}^{{\rm{T}}}+{\rm{R}}})}^{-1})}^{T}\times {(\sqrt{H{{\rm{P}}}^{{\rm{b}}}{H}^{{\rm{T}}}+{\rm{R}}}+\sqrt{{\rm{R}}})}^{-1}$$

**P**^b^ is the background-error covariance matrix, and **R** is the observation-error covariance matrix. Although it is challenging to quantify the errors both in the model simulations and in the observations, it is fairly important to estimate them accurately, since the update of the state vector depends on their relation. If the observational error variance is smaller than the model error variance, then the state vector is more strongly shifted towards the observation value; otherwise, the observation will have little impact on adjusting the model states. In ensemble-based approaches, the background-error covariances are calculated from the ensemble members^17^. No observations are free from errors. Instrumental observations have various error sources^21–23^, and estimating the error in documentary and proxy data is not straightforward either. How the errors of the different observation types are estimated is discussed in the Observational data section below. It is assumed that observational errors are uncorrelated. Hence, observational data can be assimilated one by one^9^, which further simplifies the assimilation scheme. Previous studies have shown that spurious long-range covariances can appear in the estimated **P**^b^ due to the small ensemble size. To avoid updates in the climate fields from distant observations, spatial localization is employed. Localizing **P**^b^ will limit how the observational information is distributed to all grid points as well as to the other variables in the state vector. The exact implementation of the offline EnSRF is given later in the Experimental design section.

### Atmospheric model simulations

As an a priori state, we use ModE-Sim, an ensemble of simulations using the atmospheric general circulation model ECHAM6^10^. ECHAM6 was run at T63 spectral horizontal resolution (approximately 1.8° by 1.8°). ModE-Sim is designed to sample internal variability under given boundary conditions and radiative forcings while accounting for uncertainties in these. The ensemble consists of different transient monthly-varying forcings and boundary conditions that account for uncertainties in their reconstruction^10^. For the period from 1421 to 1850, ModE-RA is based on ModE-Sim set 1420-3, consisting of 20 members that use sea-surface temperature (SST) reconstructions^24^ and climatological HadISST sea ice^25^ as boundary conditions, and PMIP4 radiative forcings^26^. For the period from 1851 to 2008, ModE-RA is based on ModE-Sim set 1850-1, which consists also of 20 members forced with HadISST2 SSTs and sea ice conditions and PMIP4 standard forcings. Concerning the mean state, ModE-Sim has the typical biases of ECHAM6 in stand-alone mode^10,27^. The most prominent difference compared to observations is a warm bias in the Northern Hemisphere mid-latitudes. Another warm bias exists over Australia, and cold biases over South America, India, and the northern Rocky Mountains. Furthermore, the model is too wet over the Himalayas and the Andes. However, despite its mean state biases, ModE-Sim is able to capture the internal variability of our key variables over land regions^10^. The performance is particularly good on seasonal-to-interannual timescales with the limitation that the ensemble spread is slightly too large in many regions^10^.

The 20 ensemble members were preprocessed by calculating the ensemble mean from the 20 ensemble members, which in the next step was transformed to 71-year monthly running climatologies (35 years before and 35 years after the current year when possible). These climatologies were then subtracted from each member to obtain the 71-year running anomalies in agreement with the assimilated anomalies. These anomalies form the *x*^*b*^ state vector in the DA framework.

### Observational data

**Fig. 2:**
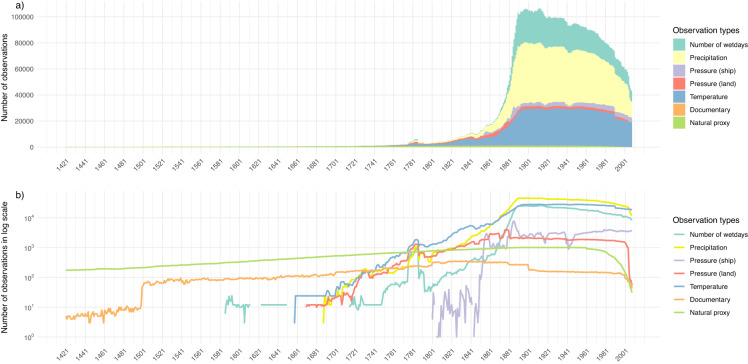
Temporal distribution of the various observation types included in the input files in absolute values per year (**a**) and in logarithmic scale (**b**).

**Fig. 3:**
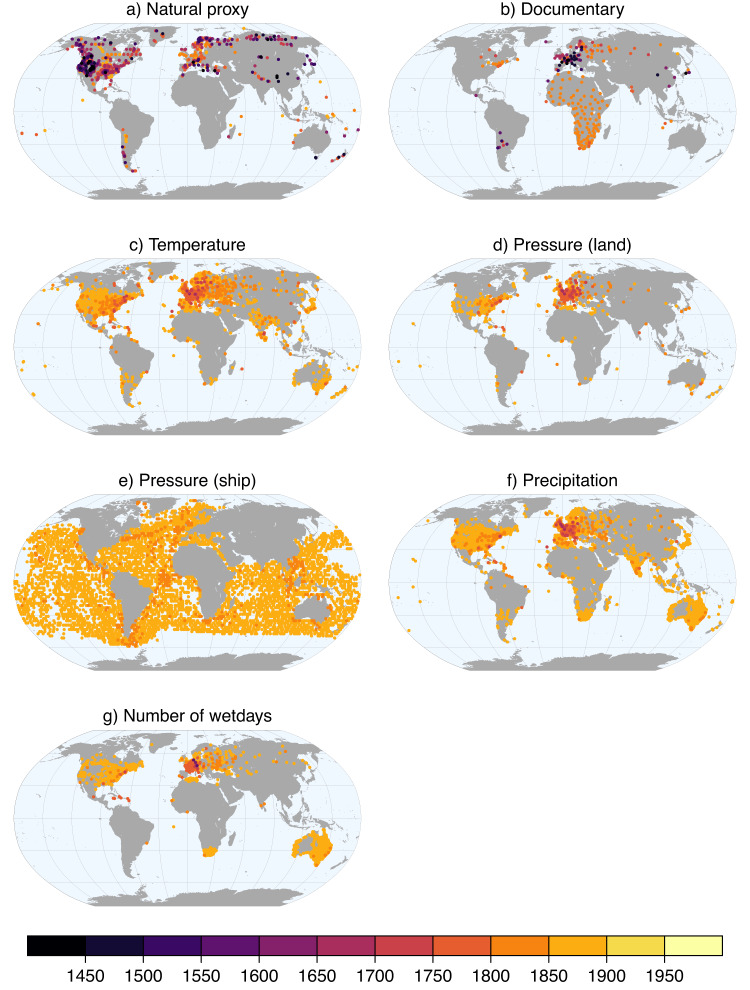
Location of observations in the input data files. Colors indicate the starting year of the time series.

In order to get the most complete information about the Earth’s climate in the last 600 years from observations, we utilize several data sources such as proxy records, documentary data, and instrumental measurements. Prior to the widespread availability of surface instrumental measurements, past climate changes can be reconstructed from proxies and documentary data. Natural proxy records are available for the full reconstruction period and instrumental measurements exist since the mid-17^th^ century. The temporal availability and spatial distribution of the different data types are shown in Figs. 2, 3.

#### Natural proxy records

Paleoclimatic proxy records provide the earliest information for our climate reconstruction from many regions of the world. Particularly useful for our focus on intra-annual to multi-decadal variability are seasonally-to-annually resolved records. The proxy network used in this study consists of several previous data compilations: PAGES2k data base^6^, OCEAN2k data base^28,29^, ISO2k data base^30^, N-TREND^31^, Briffa/Schweingruber^32^, Breitenmoser 2014^33^, and Neukom 2014^34^; and involves the assimilation of bivalve, coral, ice, lake sediment, speleothem, and tree proxy records. The PAGES2k data base contains 692 temperature-sensitive records; the major archive type is trees with ring width (TRW) as the most common variable. The OCEAN2k data base is a collection of 57 coral proxies that recorded variations in sea surface temperature. The ISO2k data base includes 759 *δ*^18^*O* and *δ*^18^*H* isotope records both from continental and marine regions. The N-TREND data base contains 54 tree-ring records from the Northern Hemisphere. The Briffa/Schweingruber dataset is based on 387 tree-ring Maximum Latewood Density (MXD) chronologies; however, we use the version which has been gridded to 5° × 5° in longitude and latitude and consists of 115 grid boxes^35^. The Breitenmoser 2014 dataset contains 2287 consistently-processed chronologies from the International Tree Ring Data Base. The Neukom 2014 dataset includes 48 marine and 277 terrestrial paleoclimatic records from the Southern Hemisphere.

In our proxy network, most records for the last millennium come from tree rings, which have one time-integrated observation per year, primarily representing the growing season. To incorporate time-integrated information into our monthly climate reconstruction, we choose a half-yearly assimilation window. We use boreal winter (October of year-1 to March), which is the Southern Hemisphere growing season, and boreal summer (April to September), which is the Northern Hemisphere growing season. The other proxy types with annual resolution have been adapted to best fit into this scheme. For instance, ice cores may record a signal during the season of strongest precipitation. Hence, we test the proxy against the climate signal from both seasons. As a result, such non-tree ring proxies can be recorders of none, one, or both seasons.

As mentioned above, we assimilate anomalies. In the first step, all proxy data are high-pass filtered by transforming them to 71-year running anomalies, i.e., each anomaly is calculated with respect to the 35 years before and 35 years after the current year when possible. This transformation is required because low-frequency variability is not retained in many TRW records whose initial goals have not been to reconstruct climate^36–38^. In the following step, all proxy records are standardized by subtracting their mean and dividing by their standard deviation. Finally, we create multiple linear regression models for each proxy, which serve as a forward operator ***H*** in the assimilation. The regression coefficients are estimated based on at least 30 years during the period 1901 to 1970 using the HadCRUT5^39^ and GPCC v2020^40^ gridded instrumental datasets for temperature and precipitation, respectively. Each month of the growing season serves as an independent variable in the regression model. We only allow for consecutive months to influence the proxies, e.g., tree-growth can depend on May-June precipitation and June-July-August temperature but not on June and August temperature without July. Based on the given information in the corresponding datasets’ publications, we apply different ***H*** forward models. See Table 2 for specific details on the proxy forward models. We test 15 temperature-only models and/or 15 precipitation-only models and/or 225 mixed (both temperature and precipitation) models per proxy record. That adds up to a theoretical maximum of 255 models (combinations of months). From these, we identify the best model based on the Akaike Information Criterion (AIC). The error of the proxy record is estimated from the regression model residuals.

**Table 2:** The possible configurations of the proxy forward operator in the different data compilations.

Date base	Temperature only	Precipitation only	Mixed
PAGES2k - tree	x		x
PAGES2k - non-tree	x		x
OCEAN2k	x		x
ISO2k	x	x	x
N-TREND	x		
Briffa/Schweingruber	x		
Breitenmoser 2014	x	x	x
Neukom 2014 - tree	x		x
Neukom 2014 - non-tree	x		x

The records in our proxy network furthermore must have a significant climate signal. Therefore, we require the p-value of the F-Test for the regression model to be statistically significant (p < 0.05) and set a threshold for *R*^2^ > 0.1. Additionally, we have visually inspected the time series of all proxy data and the match of the forward modeled proxy data. In this procedure, we identified and excluded a few records that only exhibit multi-decadal variability despite having annual values. In the final step, duplicates have been removed since proxy records can be part of several databases. Sometimes duplicates may not have identical values because there are multiple versions of the same record, for instance, after different statistical treatments like age-trend removal. Therefore, we keep the best (highest *R*^2^) and longest record within a 10 km radius.

#### Documentary data

Documentary data encompass climate information from historical documents. We use them in the form of indices (temperature, precipitation, wetness/dryness) or phenological data (flowering dates, harvest dates, river or lake freezing and thawing). Documentary data have been used for regional climate reconstructions^41–43^, but only a few records have so far been included in global datasets such as the PAGES2k data base^6^. A new global documentary data collection was published recently, combining document-based climate evidence from all around the world for the past 600 years, comprising ca. 600 series^7^. Documentary data have a unique importance in climate reconstruction because they have higher temporal resolution than natural proxy records, often monthly, and they cover combinations of seasons and regions (e.g., winter in East Asia) that are otherwise not well covered with natural proxy data. From the ca. 600 available time series in the global documentary data collection, we removed those that did not extend further back than the neighboring meteorological station that would have to be used for calibration. We assimilate phenological data such as flowering date, grape harvest date, leaf coloring date, and freezing or thawing dates of rivers or lakes (or related information such as the first and last ship in a port); specific indices such as sea ice severity, temperature or precipitation indices, and wetness or dryness indices; as well as indices that were already converted to a temperature or precipitation series. Moreover, we used various wind proxies based on marine observations of wind direction. Some of the documentary series in Europe and Asia reach back to the year 1421, to 1722 (or 1640 if we consider Guatemala rainfall index) in North America, to 1540 in South America, and to 1796 (or 1750 if we consider West African Monsoon Index) in Africa.

In order to assimilate documentary data, a forward operator (***H***) needs to be defined. Like for the natural proxies, this was done by calibrating a multiple linear regression model based on gridded climate data or station data and is described in detail in the documentary data collection paper^7,44^. All necessary details on the forward model are given in the observation feedback archive files. In short, we use monthly data of the main driving variable (either temperature or precipitation). If an index covers a fixed season or month, this season (seasonal average) or month was chosen, and the forward operator then simply extracts this month or season from the state vector. For phenological dates, we also allowed months prior to the event in question to enter the model. We therefore included all previous months back to the start of the assimilation window. In a backward selection approach, we then retained only significant months (p < 0.1) but kept insignificant months between two significant months to obtain a consistent model. That differs from Burgdorf *et al*. (2023) only insofar that in the latter paper, all six months prior to a phenological event (defined as the month in which the 90th percentile of the time series lies) were included, whereas in our approach this was restricted to the months within the six-month assimilation window of ModE-RA. As a consequence, there are a few instances in which a series (e.g., spring thawing date) appears both in the boreal winter and in the boreal summer assimilation, as both models individually are significant. Another difference was that all models were calibrated based on anomalies from the 71-year moving average, as they will be assimilated in this form. In a few cases, highly non-Gaussian variables were transformed prior to calibration^7,45,46^.

The models were calibrated in an overlapping period that was ideally 70 years long and as early as possible, but we allowed for shorter periods down to 20 years. If the overlap was too short, we standardized the documentary record and scaled it with the standard deviation of the climate field data. The regression coefficients of the final model were then used as coefficients of the forward operator, and the variances of the residuals of the regression were used as an estimation of the observational error.

#### Instrumental measurements on land

Early meteorological measurements have a key role when studying past climate variations. A new comprehensive data compilation (HCLIM), with the focus on the early instrumental period, includes global, regional, and national databases as well as various other datasets and newly digitalized time series^47^. The HCLIM database contains early instrumental time series of global air temperature measurements (3633 series) between 1658–2021, monthly global atmospheric pressure measurements (807) between 1658–2020, monthly global precipitation measurements (4944) between 1677–2021, and monthly number of wet days (3072) between 1586–2019.

The records included in the HCLIM database are quality controlled but not homogenized. However, breakpoints and merging information are provided. We used the breakpoints to prepare the instrumental measurements for the assimilation. The time series were split where breakpoints are indicated and then divided into three categories: (1) long time series, more than 50 years long, (2) medium length series, between 5 and 50 years; and (3) short time series, less than 5 years long. Therefore, while HCLIM only comprises time series starting before 1890, some of the time series resulting from the splitting will have a later starting date. We chose a simple ***H*** forward operator to assimilate the measurements, which only extracts the correct month from the state vector. In addition to the forward operator, the errors in the measurements also need to be provided. We estimated the errors following the methodology of Wartenburger *et al*.^48^. In some cases, the error had to be inflated, in particular when the monthly means were calculated as the average of the monthly extremes and when they referred to the Julian calendar. For these cases, we used nine sub-daily series from the Palatine Society network^49^ to estimate the inflation. The estimated errors of the measurements are summarized in Table 3. The long time series are transformed to anomalies with respect to 71-year moving climatologies, similar to the proxy records and documentary data, while the medium-length and short series are first left unchanged. Furthermore, if several series of the same instrumental type belong to the same grid cell in the same year, only the ones belonging to the longest category are assimilated.

**Table 3:** Estimated instrumental errors. k = (1013.25 hPa - *p*_*mean*_)/100 hPa, where *p*_*mean*_ is the average of the whole series and k is used to linearly increase the error with elevation.

Variable	Description	Error
pressure	baseline	$\sqrt{3}$ hPa
pressure	average of monthly extremes	$+\sqrt{4.7}$ hPa
pressure	Julian calendar (interpolated)	$+\sqrt{4.3}$ hPa
pressure	not reduced to sea level	$+\sqrt{1.1}$ hPa * k
temperature	baseline	$\sqrt{0.9}$ K
temperature	average of monthly extremes	$+\sqrt{1.4}$ K
temperature	Julian calendar (interpolated)	$+\sqrt{0.8}$ K
precipitation	baseline	max(10 mm, 30%)
precipitation	Julian calendar	+50%
wet days	baseline	2 days
wet days	Julian calendar	+3 days

The + indicates that an additional error is added to the baseline error.

#### Pressure measurements from ships

Marine near-surface measurements provide information from many regions where otherwise we have no other data sources. We use the International Comprehensive Ocean-Atmosphere Data Set (ICOADS) Release 3.0 to assimilate pressure measurements^50^. Nighttime marine air temperature and SST measurements have been already incorporated into the SST reconstruction^24^ used as boundary condition in ModE-Sim^10^. ICOADS is a 2° × 2° gridded dataset with monthly resolution starting in 1800. In addition to the monthly values, important metadata (e.g., number of observations per month) are also available. We use the number of observations per month to decide whether the given monthly value per grid cell is a good estimation of monthly means. The number of days with observations can come from multiple ships crossing the same grid box on the same days. However, in the earlier times, it is very unlikely that many ships crossed a grid box on the same day of the month. We set 10 observations per month as a minimum number of observations to use the data as a monthly mean. Additionally, we mask all grid cells that never met the 10 observations per month criterion at any time step until 1890. That leaves us with wide coverage because many grid cells have some observations during the 1880s. Moreover, we further thinned the amount of marine pressure measurements due to the correlated errors in the case of moving ships. In a raster of 3 × 3 grid cells, we only keep the observation at the location where the number of observations per month is the highest.

The ***H*** forward operator follows the same principle as for land measurements; that is, extracting the closest grid cell for a given month from the state vector. Observational error variances are estimated to vary between 3 hPa, which has been used for land stations in case of hundreds of observations per month and 10 hPa in the case of only 10 observations per month. We used an equation similar to what has been used previously for estimating observational error of ship measurements^24^:

5 $${\sigma }_{error}^{2}={\sigma }_{systematic}^{2}+{\sigma }_{random}^{2}/\sqrt{n}$$

where, the systematic error variance (${\sigma }_{systematic}^{2}$) is approximated with 3 hPa, the random error variance (${\sigma }_{random}^{2}$) with 22 hPa and *n* denotes the number of observations. We could not calculate the 71-yr moving climatologies because of the time gaps at the grid cells, especially in the 19th century. Instead, there is just one climatology for the entire 20^th^ century (Jan 1900 to Dec 1999). It consists of 12 values, one per month, for all grid cells. These climatologies are subtracted from the absolute values to create the anomalies for the assimilation.

### Experimental design

In our DA framework we work with anomalies. As already described above, both the model simulations (***x***^*b*^) and long observations (***y***) are transformed into anomalies, except for the ship pressure measurements, where anomalies are calculated relative to the 1900–1999 climatology. Since we only assimilate anomalies, the centennial-scale variability in our dataset is influenced by the reaction of the model to prescribed forcings, whereas annual-to-multi-decadal variability is improved based on the assimilated observations. By working with anomalies, we do not correct for existing biases in the model simulations and the reconstructions remain consistent in the model world. This has the advantage of not introducing artificial trends due to temporal changes in the observation data availability.

The implementation of offline DA approaches and the estimation of the background-error covariance matrix vary among the different paleoclimate reconstructions. One offline DA method assumes a time-invariant **P**^**b**^^1,3^, while in others **P**^**b**^ is forcing-dependent (e.g., amount of volcanic aerosols, ENSO state, etc.) and is recalculated from the precomputed model ensemble for this specific time step^2^. In a further study, time-invariant and transient covariance matrices are combined to offset the relatively small sample size of transient simulations and thereby obtain a better estimation of the errors^5^. Here, we also use a blending technique to calculate the background errors to compensate for the small ensemble size. The blended background errors (**P**^**blend**^) are calculated by combining a climatological background-error covariance matrix **P**^**clim**^ with the transient one (**P**^**b**^) as:

6 $${{\bf{P}}}^{{\bf{blend}}}={\beta }_{1}{{\bf{P}}}^{{\bf{b}}}+{\beta }_{2}{{\bf{P}}}^{{\bf{clim}}}$$

where **P**^**clim**^ is derived by randomly selecting 100 climate states from the 590 year-long transient model simulations (20 ensemble members * 590 years) for each assimilation window. Based on previous results^51^ the weights (*β*_1_, *β*_2_) of the covariance matrices set to be equal. Both *β*_1_ and *β*_2_ are 0.5. Hence, in our DA scheme, the **P**^**b**^ is replaced with **P**^**blend**^ in Eqs. 3, 4.

As mentioned above, the impact of an observation on ***x***^*b*^ is often limited by localizing the background-error covariance matrix. We implement a widely used localization technique, where each element of the error covariance matrices is multiplied with the respective element of a distant-dependent correlation function. We use a Gaussian localization function as:

7 $$G={\exp }\left(-\frac{{z}^{2}}{2{L}^{2}}\right)$$

where the distance between two grid boxes is denoted by *z*, and *L* stands for the length-scale parameter. We apply a stricter localization on **P**^**b**^, which is calculated from 20 ensemble members than on **P**^**clim**^, which we calculate from 100 members. The applied length-scale parameters are summarized in Table 4. We also set values to 0 where the distant-dependent correlation is <0.001.

**Table 4:** Length-scale parameters used in the localization of **P**^b^ and P^clim^ matrices. The values are given in km.

Variable	P^b^	P^clim^
temperature at 2 m	1500	3000
sea-level pressure	2700	5400
precipitation	450	900
wet days	450	900
u10	500	1000
v10	500	1000

In order to be able to track the impacts of observations, we provide an observation feedback archive in which metadata and information about the preparation of the input data are given. The metadata and preparation information are the following: observation and record ID, the name, the coordinates, as well as the altitude. Additionally, we provide the year in which the observation was taken, the year in which it is assimilated, the season, the data type, variable and the unit, the original and transformed value (e.g., interpolation from Julian to Gregorian calendar in early instrumental data or standardization in case of documentary and proxy data,) as well as the 71-year moving climatology and anomaly, the reference period and reference dataset, the inherited quality check flags, the observational error variance, to which cycle the observation belongs and the coefficients of the ***H*** forward operator. Regarding the assimilation, for each record we store the flags of the background check and whether the record is assimilated, the length-scale parameters, the background and analysis departure in the same grid cell from each member, the ensemble mean and from the 71-year model climatology, and a bias term of medium and short observations.

In our setup ***H*** is always linear and when necessary calibrated in the preprocessing steps and stored in the half-yearly (previous year’s October to March and April to September) observational input files together with the observational error variances. The ***x***^***b***^ state vector is built from 6 monthly fields of several variables. Therefore, we not only apply a localization in space but also in time; that is, we allow the observations to update the monthly fields based on the ***H*** forward operator. In the case of monthly instrumental measurements, these will have an influence only on one month. Seasonal documentary and proxy data, however, can affect several months. After preparing the observations and the monthly anomalies of the ensemble members, in the first step we assimilate all proxy records, documentary data and marine measurements as well as long (>50 years) instrumental measurements. Before a non-marine observation is assimilated we check first whether the observation is within the ±5 range of the square root of the sum of model and observation variances $\left(\sqrt{{\boldsymbol{H}}{{\bf{P}}}^{{\bf{b}}}{\boldsymbol{H}}+{\bf{R}}}\right)$ from the model ensemble mean. In the case of marine observations a stricter filtering was implemented because we wanted to give less weight to pressure measurements from ships. Therefore a marine sea-level pressure record has to be within a ±2 range of the model ensemble mean. If an observation does not pass the prescribed quality check, it is assimilated passively; that is, the climate state is not updated by the observation but its potential impact is stored in the observation feedback archive file. Furthermore, instrumental measurements of the same type are averaged if more than one observation can be found in the same grid box, and then their average is assimilated.

The assimilation is paused after assimilating all proxies, documentary data, and instrumental records longer than 50 years in the first cycle (cycle1). The 5 to 50 year-long instrumental time series are debiased before they are assimilated in the second cycle (cycle2) (Fig. 1). For debiasing we use a simple approach of fitting the first two harmonics to the annual cycle of the observation and the ensemble mean of cycle1 analysis. The annual cycles of the cycle1 analysis and the medium-length time series (belonging to cycle2) are calculated from the months where measurements are available. In most cases there is a good agreement between the annual cycle of the cycle1 analysis and the medium-length observations in the temperature and pressure time series, and the harmonic fits approximate well the annual cycles. The annual cycles of number of wet days and precipitation calculated from the cycle1 analysis and the observations agree less, and the harmonic fits follow less the month-to-month variations. In cycle2, the time series are transformed to anomalies as:

8 $${x}_{anom}={x}_{orig}-bias-mode{l}_{clim}$$

where *x*_*orig*_ is the original time series, bias is the monthly bias between the fitted annual cycles and *model*_*clim*_ is the 71-year running climatology of the model. From this point, the observation goes through the same steps (quality check, averaging) as in cycle1, before it is assimilated. In cycle2 the model simulations are replaced with cycle1 analysis as the background state and the covariance matrices are also recalculated from them. After assimilating all observations in cycle2, we pause again the assimilation and the biases in the short records (<5 years) are calculated similarly as for cycle2 observations, fitting the harmonics to the ensemble mean of cycle2 analysis and the observations. Then using Eq. 8 the short records are transformed to anomalies and assimilated. The cycle3 analysis is the final product, the ModE-RA paleo-reanalysis.

In addition to the ModE-RA paleo-reanalysis, we generated another reanalysis ModE-RAclim to allow the user to disentangle the effect of observations and boundary conditions (forcings) on ModE-RA. For ModE-RAclim the members of the ***x***^*b*^ state vector are randomly selected from the 589 year-long 20 transient ModE-Sim simulations. We use 100 randomly selected members at each half year in the assimilation from which the **P**^**b**^ background-error covariance matrix is calculated. This approach is similar to other reconstructions^1,3^ with the exception that they always use the same ***x***^*b*^ and **P**^**b**^ and work with absolute values. Note that the random sampling used for the ModE-RAclim prior largely averages out the forced signal arising from the forcings and boundary conditions in ModE-Sim and the assimilated observations have been high-pass filtered. Variability at scales longer than 71 years is not present in this sensitivity experiment. For the localization of the background-error covariance matrix the larger length-scale parameters were employed (Table 4, right column). Observations are handled as in the ModE-RA assimilation, assimilating them in three cycles.

## Data Records

Most of the input datasets are publicly available and can be downloaded from the following data repositories: PAGES2k records can be found on figshare^52^, Ocean2k^53^ and Iso2k^54^ can be downloaded from NOAA/WDS Paleoclimatology; the N-TREND dataset can be found on the project website^55^; the gridded Briffa/Schweingruber tree-ring dataset can be downloaded from the Climatic Research Unit^56^; the processed tree-ring chronologies by Breitenmoser 2014 were used in previous climate reconstruction and are available within the EKF400 project^57^; DOCU-CLIM is accessible on BORIS^58^; HCLIM can be downloaded from PANGAEA^59^; ICOADS data provided by the NOAA PSL, Boulder, Colorado, USA, from their website (https://psl.noaa.gov). The record used from the Neukom 2014 dataset is included in the observation feedback archive and uploaded to WDCC (10.26050/WDCC/ModE-RA_s14203-18501).

## Technical Validation

The quality of the ModE-RA paleo-reanalysis is assessed by comparisons with multiple data sources, depending on their availability through time. We start with comparisons to gridded instrumental datasets in the 20^th^ century. Because these are not fully independent and the quality of ModE-RA changes through time, we additionally compare ModE-RA to the 20^th^ Century Reanalysis version 3^60^ (20CRv3) and mostly proxy based reconstruction^1,3,4,42^. Finally, we compare ModE-RA to completely independent documentary information. As metrics for evaluation, we use the correlation and the root mean square error skill score (RMSESS). The RMSESS is calculated as:

9 $$RMSESS=1-\frac{\sqrt{\frac{1}{n}{\sum }_{i=1}^{n}{\left({x}_{i}^{u}-{x}_{i}^{ref}\right)}^{2}}}{\sqrt{\frac{1}{n}{\sum }_{i=1}^{n}{\left({x}_{i}^{f}-{x}_{i}^{ref}\right)}^{2}}}{\rm{,}}$$

where we replace ***x***^*u*^ with the ensemble mean of the paleo-reanalysis (ModE-RA) (${\bar{x}}^{a}$); ***x***^*f*^ is the ensemble mean of the model simulations (ModE-Sim), i.e., this skill score measures the improvement in comparison to ModE-Sim, which already has some reconstruction skill because it follows the external forcings and boundary conditions. (${\overline{x}}^{b}$). The time step is denoted with *i*; we calculate the RMSESS over the 1901–2000 period. For the 20^th^ century validation, we compare the ensemble mean of ModE-RA against three reference datasets for temperature, precipitation, and sea level pressure (***x***^*ref*^): HadCRUT5^39^, GPCC version 2022^14,61^, and HadSLP2^62^. These datasets are not entirely independent from the paleo-reanalysis, but only time series starting before 1890 (before the breakpoint detection) were assimilated in ModE-RA. The skill metrics are calculated using anomalies relative to the 1961–1990 period. All three validation datasets were remapped to the spatial resolution of the model simulations. Although ModE-RA has monthly resolution, we present half-year averages in the evaluation because of similar absolute values and very small differences between single months (see Figs. S1–S3). Differences between the two half-years are more relevant because many proxies, which represent a growing season, have only been assimilated in one of the half-year assimilation windows.

**Fig. 4:**
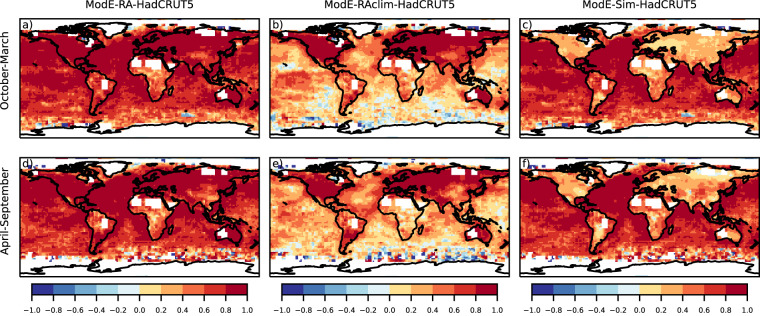
Correlation of seasonal mean temperature: Correlation is calculated between the ensemble mean of ModE-RA temperature and the HadCRUT5 reference dataset (**a,d**), between the ensemble mean of ModE-RAclim temperature and HadCRUT5 (**b,e**), and between the ensemble mean of ModE-Sim temperature and HadCRUT5 (**c,f**). Correlations are calculated over the 1901–2000 time period and all datasets (except ModE-RAclim) are transformed to anomalies relative to the 1961–1990 climatologies. ModE-RAclim is used without adding back any kind of climatology.

**Fig. 5:**
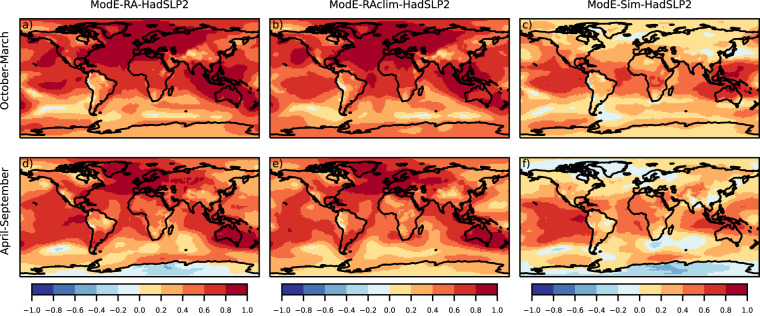
Correlation of seasonal mean sea-level pressure: Correlation is calculated between the ensemble mean of ModE-RA sea-level pressure and the HadSLP2 reference dataset (**a,d**), between the ensemble mean of ModE-RAclim sea-level pressure and HadSLP2 (**b,e**), and between the ensemble mean of ModE-Sim sea-level pressure and HadSLP2 (**c,f**). Correlations are calculated over the 1901–2000 time period and all datasets (except ModE-RAclim) are transformed to anomalies relative to the 1961–1990 climatologies. ModE-RAclim is used without adding back any kind of climatology.

**Fig. 6:**
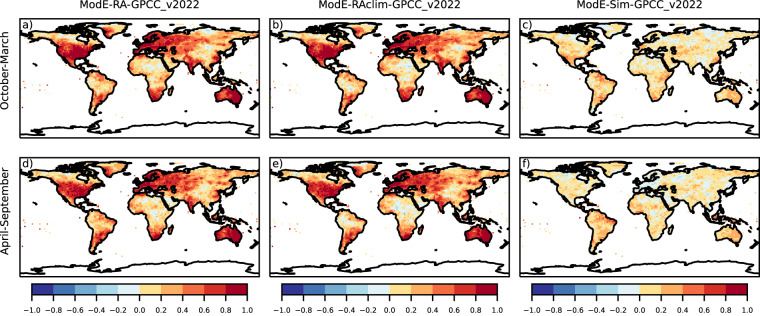
Correlation of seasonal mean precipitation: Correlation is calculated between the ensemble mean of ModE-RA precipitation and the GPCC version 2022 reference dataset (**a,d**), between the ensemble mean of ModE-RAclim precipitation and GPCC version 2022 (**b,e**), and between the ensemble mean of ModE-Sim precipitation and GPCC version 2022 (**c,f**). Correlations are calculated over the 1901–2000 time period and all datasets (except ModE-RAclim) are transformed to anomalies relative to the 1961–1990 climatologies. ModE-RAclim is used without adding back any kind of climatology.

Temperature correlation between ModE-RA and gridded instrumental dataset are globally high (Fig. 4a,d). Note that the HadCRUT5 dataset is a blended product and uses the HadSST4 sea-surface temperature dataset^63^ which is also one of the boundary conditions of ModE-Sim; therefore the high correlation values over oceans are expected (Fig. 4c,f). Correlations are also mainly positive over land between ModE-Sim and HadCRUT5 due the 20^th^-century warming trend, but after assimilating the observations an almost perfect correlation is achieved especially in the Northern Hemisphere and Australia (Fig. 4a,d). For pressure, ModE-Sim has the strongest correlation with HadSLP2 over the tropical ocean (Fig. 5c,f). The assimilation improves the sea-level pressure fields the most over the Northern Hemisphere, in southern South America and a larger region around Australia (Fig. 5a,d). In contrast to temperature and sea level pressure, where the forcings and boundary conditions introduced positive correlations in ModE-Sim, correlation coefficient of ModE-Sim for precipitation are close to zero (Fig. 6c,f). Hence, most information is gained by the assimilation (Fig. 6a,d). Correlation coefficients of precipitation improved in the regions, where most of the precipitation data were assimilated because of the narrower localisation length scale (Table 4).

In addition to analyzing the performance of ModE-RA, the skill of ModE-RAclim is also shown on the figures (Figs. 4b,e, 5b,e, 6b,e). The reconstructed temperature fields of ModE-RAclim have similar skill over land where the skill comes from data assimilation. ModE-RA outperforms ModE-RAclim over marine regions where the skill comes from the SST boundary conditions. The results of the sea-level pressure reconstruction of ModE-RAclim yields again similar skills to ModE-RA, but ModE-RA has higher skill over the tropical Pacific where the information comes again from the SST boundary conditions. The skill of the ModE-RAclim precipitation field is comparable to ModE-RA because ModE-Sim has correlations close to zero and most information is gained by the assimilation.

**Fig. 7:**
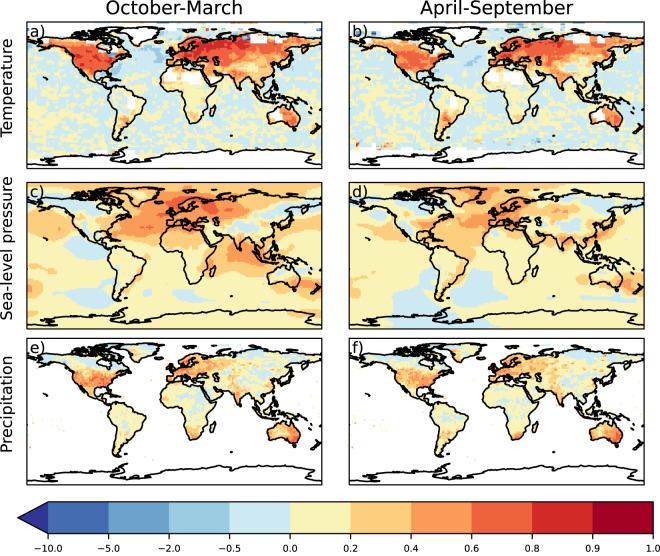
RMSESS of seasonal mean temperature (**a,b**), sea-level pressure (**c,d**), and precipitation (**e,f**). RMSESS is calculated over the 1901–2000 time period using the ModE-RA ensemble mean, the model ensemble mean and the reference datasets of HadCRUT5, HadSLP2, GPCC version 2022; all transformed to anomalies relative to the 1961–1990 climatologies.

To evaluate the skill of ModE-RA compared to the model simulation, the RMSESS is calculated by using the ensemble mean of ModE-Sim in the denominator (***x***^*f*^ in Eq. 9). With the help of the RMSESS metric we can gain further insights into how well the amplitudes of climate variability are reconstructed. The temperature fields show a notable improvement over ModE-Sim in the extra-tropical land areas of the Northern Hemisphere, the southern part of South America, and Australia (Fig. 7a,b). Sea-level pressure is better reconstructed in the October to March half year than in April to September. The largest increases in the RMSESS can be found over the North Atlantic, Europe, and the Indian Ocean (Fig. 7c). The reconstructed precipitation fields show improved skill mainly in the regions where precipitation measurements were assimilated in both half years (Fig. 7e,f).

**Fig. 8:**
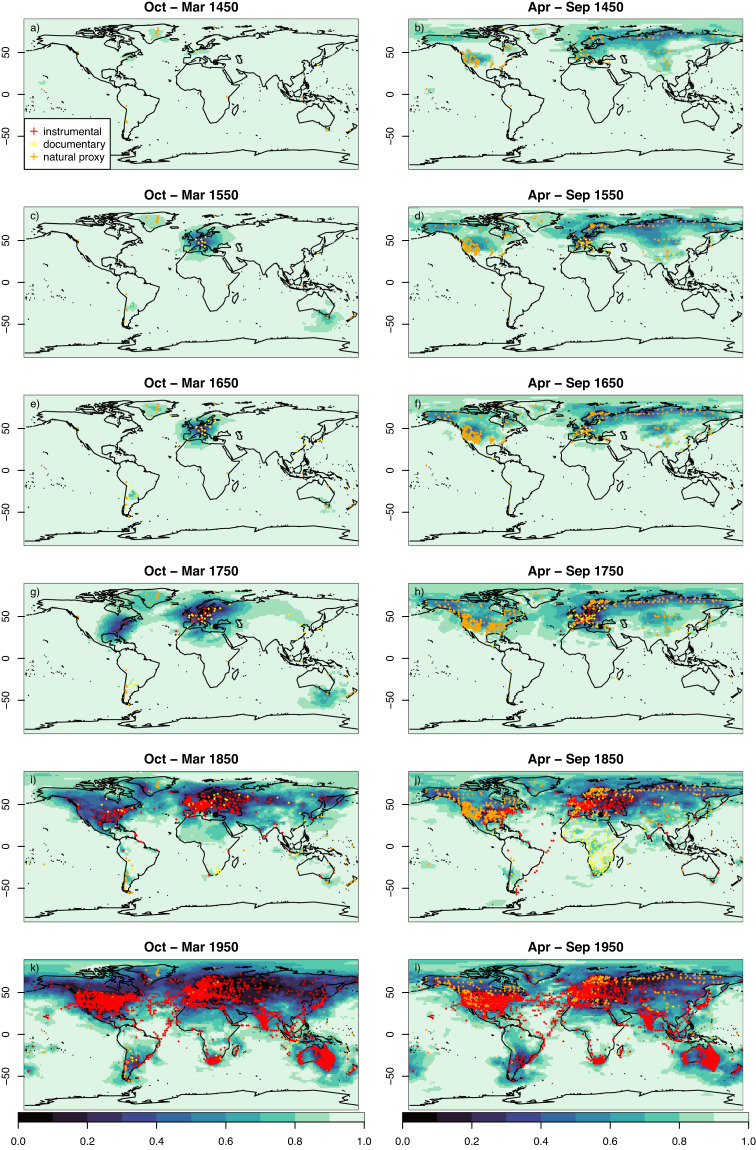
Ensemble standard deviation ratio (ModE-Sim/ModE-RA) of temperature, i.e., the remaining ensemble standard deviation after data assimilation in comparison the the prior standard deviation. A value of one indicates no information added by data assimilation and a value of 0 would indicate that no ensemble spread is left. This is shown in the color scale for January (left column, (**a**)), (**c**), (**e**), (**g**), (**i**), (**k**)) and July (right column, (**b**), (**d**), (**f**), (**h**), (**j**), (**l**)) for 6 time slices between 1450 and 1950. The symbols indicate the assimilated observation network for all variables in the corresponding October to March (left column) and April to September season (right column) of the same year.

The 20^th^ century validation highlights the quality of ModE-RA at the end of the 19^th^ century because no stations that started measuring after 1890 were included anymore. As seen in Fig. 2, the number of assimilated observations decreases back in time and simultaneously the spatial coverage and density shrinks. The ensemble standard deviation ratio (standard deviation of the ModE-Sim ensemble divided by the standard deviation of the ModE-RA ensemble) highlights where the assimilation added information. This remaining ensemble standard deviation after data assimilation in comparison to the prior standard deviation for six time slices between 1450 and 1950 and for two months is shown in Fig. 8 (January: a,c,e,g,i,k and July: b,d,f,h,j,l). There are few observations assimilated in the October to March season before the year 1500. First observations for the boreal winter season become available in the 16^th^ century. In the 18^th^ century, the spatial distribution extends into eastern North America. In contrast, there is already a dense proxy network in the northern hemisphere extra-tropical land region during the April to September season in the year 1450. This is also further increasing through time. Assimilated information in the Southern hemisphere before the year 1800 is sparse. Nevertheless, ModE-RA includes more observations from the 19^th^ and early 20^th^ century than any other currently existing gridded global dataset for data sparse regions such as South America or Africa.

To further evaluate ModE-RA beyond the 20^th^ century, we compare regional-scale variations of the ModE-RA ensemble mean with other reconstructions. Several annual global temperature field reconstructions based on natural climate proxies exist over the Common Era. In a recent study, annual global temperature was generated with six different climate field reconstruction techniques (Analogue method (AM), Canonical correlation analysis (CCA), Composite plus scaling (CPS), Data assimilation (DA), GraphEM (GEM), and Principal-Component Regression (PCR)), all using the same multiproxy input data^6^ over the Common Era^4^. Additionally, we compare ModE-RA to the global temperature reconstructions with annual resolution from the Last Millennium Reanalysis (LMR), produced with a similar offline DA method and assimilating multiproxy records^1^. A reconstruction of paleo-hydroclimate (PHYDA) - reconstructed with an offline DA method using proxy records - provides both annual and seasonal climate fields of several variables^3^. Furthermore, a monthly temperature reconstruction for European land areas is also available starting in 1659, which is based on multiproxy records, documentary evidence, and instrumental data (hereafter L2004^42^).

**Fig. 9:**
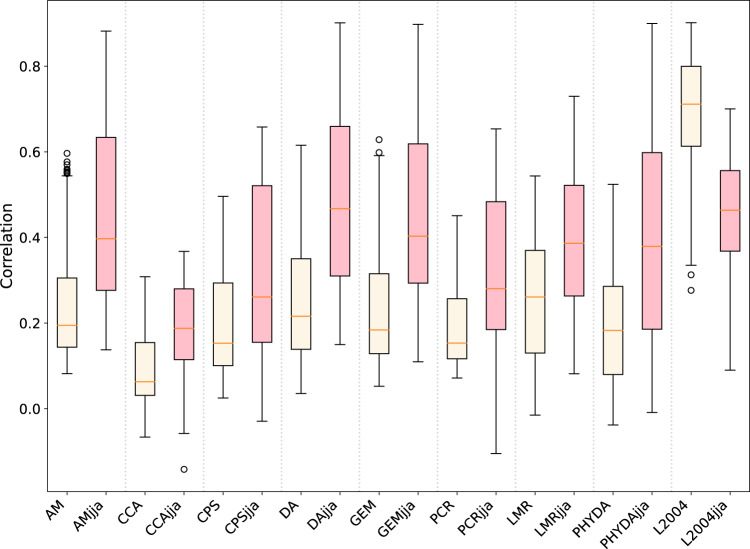
Annual and JJA seasonal correlation values of temperature over the European land area. Correlation is calculated for each grid cell in the overlapping period between the annual temperature reconstructions (AM, CCA, CPS, DA, GEM, PCR, LMR, PHYDA) or in the case of higher temporal resolution, first annual means are calculated (L2004) and the annual mean temperature of the ensemble mean of ModE-RA (yellow). Additionally, correlation is calculated between the annual reconstructions (AMjja, CCAjja, CPSjja, DAjja, GEMjja, PCRjja, LMRjja) or JJA mean values when possible (PHYDAjja, L2004jja) and the JJA mean temperature of the ensemble mean of ModE-RA (pink). The lower and upper borders of the boxes represent the first quartile and the third quartile of the data, respectively. The orange midlines of the boxes are the medians. The whiskers extend from the box by 1.5 times the inter-quartile range.

For the pre-20^th^ century, we focus on the European land area (25°W–40°E and 35°N–70°N, as defined in L2004). The correlation was calculated at each grid cell between the reconstructions and the ensemble mean of ModE-RA in the overlapping period after the reconstructions were remapped to the resolution of ModE-RA. In the six reconstructions^4^ a year is defined as April to March, similar to the annually resolved PHYDA reconstruction^3^. Therefore, annual values from ModE-RA and L2004^42^ were calculated correspondingly from the monthly April to March data. Correlation between LMR and ModE-RA in the annual comparison is calculated between January and December. All annual reconstructions based on multiproxy records mainly use information from tree rings. These primarily provide information for the summer season. Hence, we calculated the correlation between the annual reconstructions and seasonal temperature field from ModE-RA using the months June, July and August (JJA). When the temperature reconstructions are available with higher temporal resolution such as in the case of PHYDA and L2004, we use them to calculate the seasonal correlation. Annual mean correlations between ModE-RA and the other reconstructions are rather low with the exception of L2004 (Fig. 9). When the correlations are calculated between the annual reconstructions (AM, CCA, CPS, DA, GEM, PCR, LMR) and the seasonal summer mean of ModE-RA, the correlation coefficients are higher (Fig. 9), suggesting that previous annual temperature reconstructions are biased towards the summer seasons. The correlation is higher between the boreal summer PHYDA reconstruction and ModE-RA than the annual reconstruction. In contrast, L2004 shows weaker correlations in the boreal summer than in the annual mean comparison (Fig. 9). The reason behind a weaker correlation in boreal summer may be that the annual mean is dominated by winter variability.

Only few datasets have monthly or higher resolved information in the period before the year 1900. Besides L2004, 20CRv3 spans over the 1836–2012 time period and was generated with a DA technique using pressure observations and a weather forecast model^60^. A more experimental phase of 20CRv3 between 1806 and 1835 is also available. Here, we use L2004 and 20CRv3 to compare the area-weighted monthly temperature anomalies, i.e., after removal of the annual cycle and relative to 1821–1850 over the European land areas in the preindustrial period. The correlation coefficient between ModE-RA and L2004 European average temperature over the 1659–1850 period is 0.27, and over the 1806–1850 period is 0.28. The correlation between ModE-RA and 20CRv3 over the 1806–1850 period is 0.67. We do not expect high correlations because L2004 is a reconstruction based on a smaller dataset with a purely statistical principal component regression method and in 20CR only surface pressure is assimilated and no temperature information.

**Fig. 10:**
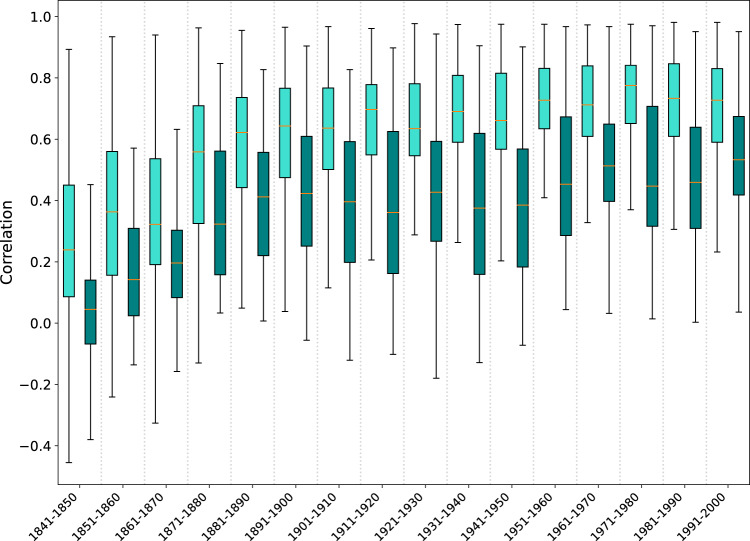
Correlation between the 500 hPa geopotential height of the ModE-RA ensemble mean and the 550 hPa geopotential height of 20CRv3. The correlations are calculated based on monthly values for each grid cell per decade, separately for the Northern Hemisphere (light green) and the Southern Hemisphere (dark green). ModE-RA and 20CRv3 are transformed to anomalies relative to the 1961–1990 climatologies. Note that outliers are not shown.

Additionally, we calculated the correlation between the 500hPa geopotential height in the ModE-RA ensemble mean and the 550hPa geopotential height in 20CRv3 from 1841 to 2000 for each decade. These fields are only updated through the data assimilation framework because no upper air observations were assimilated. The correlations are calculated based on monthly values relative to the 1961–1990 climatologies, separately for the Northern and the Southern Hemisphere using the ensemble means. In general, there is better agreement between the ModE-RA and 20CRv3 over the Northern Hemisphere with the median of correlations strongly increasing until the 1911–1921 decade then the changes become relatively small from decade to decade (Fig. 10). The agreement between ModE-RA and 20CRv3 in the Southern Hemisphere increases throughout the examined period with a slowdown from 1901 to 1950.

**Fig. 11:**
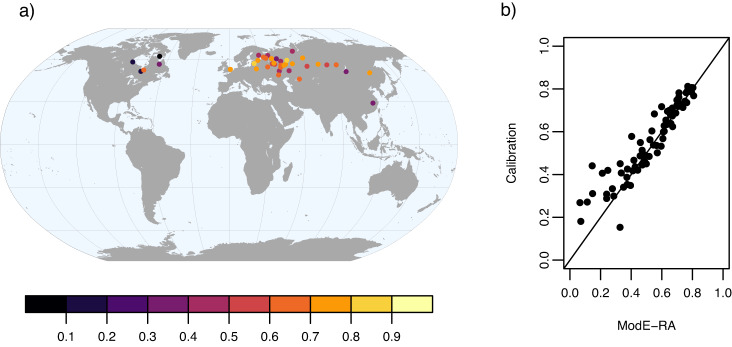
Correlation between independent documentary data and the ensemble mean of ModE-RA (**a**). Note that sometimes several series are available from the same locations and in that case only the highest value can be seen on the map. The correlation of forwards models in the calibration process and when ModE-RA is used closely fit to the 1:1 line (**b**).

Furthermore, we used 71 independent documentary records (Table S1) from the documentary data compilation^7^ to evaluate ModE-RA in the earlier times. The independent documentary data are first calibrated with observation-based datasets as described in documentary data compilation paper^7^, and then these models are applied to ModE-RA for estimating the documentary observations. We found strong correlations between the independent data and the ensemble mean of ModE-RA, except for a few series, especially in North America (Fig. 11a). Comparing the correlations of ModE-RA with the calibration statistics, they are almost on a 1:1 line (Fig. 11b).

The observation feedback archive can also be used to analyze individual cases. Here, we present three examples of extremely positive and negative Palmer drought severity indices (PDSI)^64^ calculated using the temperature and precipitation fields from ModE-RA. The PDSI calculation requires three inputs: potential evapotranspiration (PET), precipitation, and latitude. We estimate PET with the Thornthwaite equation^65^, which is solely based on monthly mean temperature, in our case from ModE-RA. This PET estimate is then used together with monthly mean precipitation sums to calculate PDSI. The original version of PDSI was calibrated to conditions in central North America. Here, we use the self-calibrating version of PDSI, which adjusts parameters in the PDSI calculations to local conditions around the world^66^. We compared the PDSI of ModE-RA with two mainly tree-ring based reconstructions, with PHYDA^3^ and the Old World Drought Atlas (OWDA)^67^. All three examples represent the boreal summer season.

1. In the summer 1789 in Central Europe, the PDSI values are negative in PHYDA and OWDA, while they are positive in ModE-RA. In the observation feedback archive we found that assimilated early instrumental precipitation measurements and information about the number of wet days per month for the region around 9°E and 49°N point to a strong wet anomaly. This is confirmed by a negative pressure anomaly in early instrumental data. In this case, there is strong support that ModE-RA can be trusted.
2. In the year 1780, ModE-RA shows a strong wet anomaly in the region of Inner Mongolia, around 101° E, 40° N. In the observation feedback archive we found only one precipitation-sensitive tree-ring record in the area at that time. It turns out that the strong positive precipitation anomaly, as well as a strong cold anomaly, are already present in ModE-Sim, which was not significantly modified by the assimilation of this single tree-ring record. We could not identify an external forcing which could be responsible for this anomaly. One possible explanation could be that all members of our relatively small ensemble are coincidentally too moist in this case. Such insights can be gained from the comparison of the model simulations with the paleo-reanalysis.
3. Finally, a drought in summer 1540 in Central Europe is reconstructed by both the OWDA and PHYDA, although with different intensities. PHYDA suggests a moderate drought whereas OWDA points to more extreme conditions. In ModE-RA, we assimilated documentary data in addition to the tree-ring proxies. These documentary indices show strong negative precipitation anomalies and positive temperature anomalies^68^. Due to the assimilation of this additional documentary evidence, ModE-RA PDSI supports the stronger drought conditions reconstructed by the OWDA.

## Usage Notes

ModE-RA (ensemble members and statistics) and ModE-RAclim (ensemble statistics) are uploaded to NOAA and to the World Data Center for Climate (WDCC) at Deutsches Klimarechenzentrum in Hamburg, Germany (10.26050/WDCC/ModE-RA_s14203-18501 and 10.26050/WDCC/ModE-RAc_s14203-18501)^69^. The two climate reconstructions are in NetCDF4 format; the NetCDF4 files cover the whole period per variable. Ensemble statistics include the mean (monthly anomalies with respect to the period 1901 to 2000), maximum, minimum and spread in terms of one standard deviation from the ensemble mean. The observation feedback archive files are available in tsv format (one file per 6 months), which contain all relevant information of the input data, how the input data were processed, and useful feedback information from the DA system. A detailed list of all information stored in the feedback archive has been published with the dataset: https://www.wdc-climate.de/ui/entry?acronym=ModE-RA_info. We provide an online visualisation tool to plot maps, timeseries and the locations of assimilated data: http://mode-ra.giub.unibe.ch/climeapp.

The ModE-RA paleo-reanalysis is identical to the ModE-Sim simulations^70^ in areas far away from any assimilated observations, especially at the beginning of the reconstruction period. With time, more and more observations are available, suggesting that the reconstruction becomes more skillful. Therefore, the users first should ensure how reliable the paleo-reanalysis is for a given region and time period. This can be achieved by looking at the ensemble spread and the differences between ModE-Sim and ModE-RA. Among the reconstructed variables, the ones with observational input data are the most realistically estimated. We encourage the users to make use of the ensemble members and not only the ensemble mean.

ModE-Sim was generated in two phases (1420–1850 and 1850–2009) with different boundary conditions^10^. In the earlier period, ModE-RA is based on ModE-Sim Set 1420-3, and in the later period on ModE-Sim Set 1850-1. ModE-RA is not split into the two periods of the ModE-Sim prior because the assimilated observational time series lead to a smooth transition between the two periods of the ModE-Sim sets.

ModE-RA was generated by transforming both model simulations and observations to 71-year running anomalies. Hence, users should be aware that the centennial-scale variability is the model response to forcings. Therefore, we see great potential for future research, particularly in terms of intra-annual to multi-decadal variability. We provide monthly anomalies with respect to the 1901 to 2000 climatology and the model climatology for the 1901 to 2000 period. Be aware that the model climatology includes model biases. Therefore, we recommend using anomalies instead of absolute values.

Furthermore, because of the employed setup, unrealistic values (such as negative precipitation) can occur if absolute values are generated by adding back a climatology. This is especially an issue in arid regions where monthly precipitation is not normally distributed. Precipitation is consistent in the periods of 1421–1800 and 1900–2009 when the observational network is quite stable, but in the 19^th^ century, when many of the observation time series start, a trend is introduced in some arid land regions and tropical oceans (Fig. S4). Hence, in the case of the reconstructed precipitation fields, the early and late period should be looked at separately.

ModE-RAclim should be seen as a sensitivity study and is only a side product of the project. ModE-RAclim does not contain centennial scale climate variability. For most users, the main product ModE-RA therefore should be used for regular studies on past climate. The main differences between ModE-RAclim and ModE-RA are on the model side: ModE-RAclim uses 100 randomly picked years from ModE-Sim as a priori state. Thereby, stationarity in the covariance structure is assumed, and the externally-forced signal in the model simulations is eliminated. In combination with ModE-Sim and ModE-RA it can be used to distinguish the forced and unforced parts of climate variability seen in ModE-RA.

ModE-RA makes use of several data compilations and assimilates various direct and indirect sources of past climate compared to 20CRv3. Hence, if monthly resolution is sufficient for the planned study, ModE-RA may have higher quality already from 1850 backwards to analyze past climate changes and can be viewed as the backward extension of 20CRv3.

### Supplementary information

Supplementary Information

## Data Availability

The R code for the quality control of the observational data and their assimilation ModE-RA ensemble can be found together with the entire ModE-RA dataset and observation feedback archive at the World Data Center for Climate (WDCC) at Deutsches Klimarechenzentrum in Hamburg, Germany (10.26050/WDCC/ModE-RA_s14203-18501).
