# Supplementary material for: ModE-RA: a global monthly paleo-reanalysis of the modern era 1421 to 2008
Source: Sci Data. 2024 Jan 5;11:36. doi: 10.1038/s41597-023-02733-8 (PMC10770343; doi:10.1038/s41597-023-02733-8)
Supplement: Supplementary file 1 — Supplementary Information [file 41597_2023_2733_MOESM1_ESM.pdf]

Supplementary Information: ModE-RA - a global monthly paleo-reanalysis of the modern era (1421-2008)

Veronika Valler[1,2,], Jörg Franke[1,2,*,**], Yuri Brugnara[1,2], Eric Samakinwa[1,2], Ralf Hand[1,2], Elin Lundstad[1,2], Angela-Maria Burgdorf[1,2], Laura Lipfert[1,2], Andrew Ronald Friedman[1,2], and Stefan Brönnimann[1,2]**

[1]Oeschger Centre for Climate Change Research, University of Bern, Bern, Switzerland
[2]Institute of Geography, University of Bern, Bern, Switzerland
[*]corresponding author(s): Jörg Franke (joerg.franke@unibe.ch)
[**]these authors contributed equally to this work

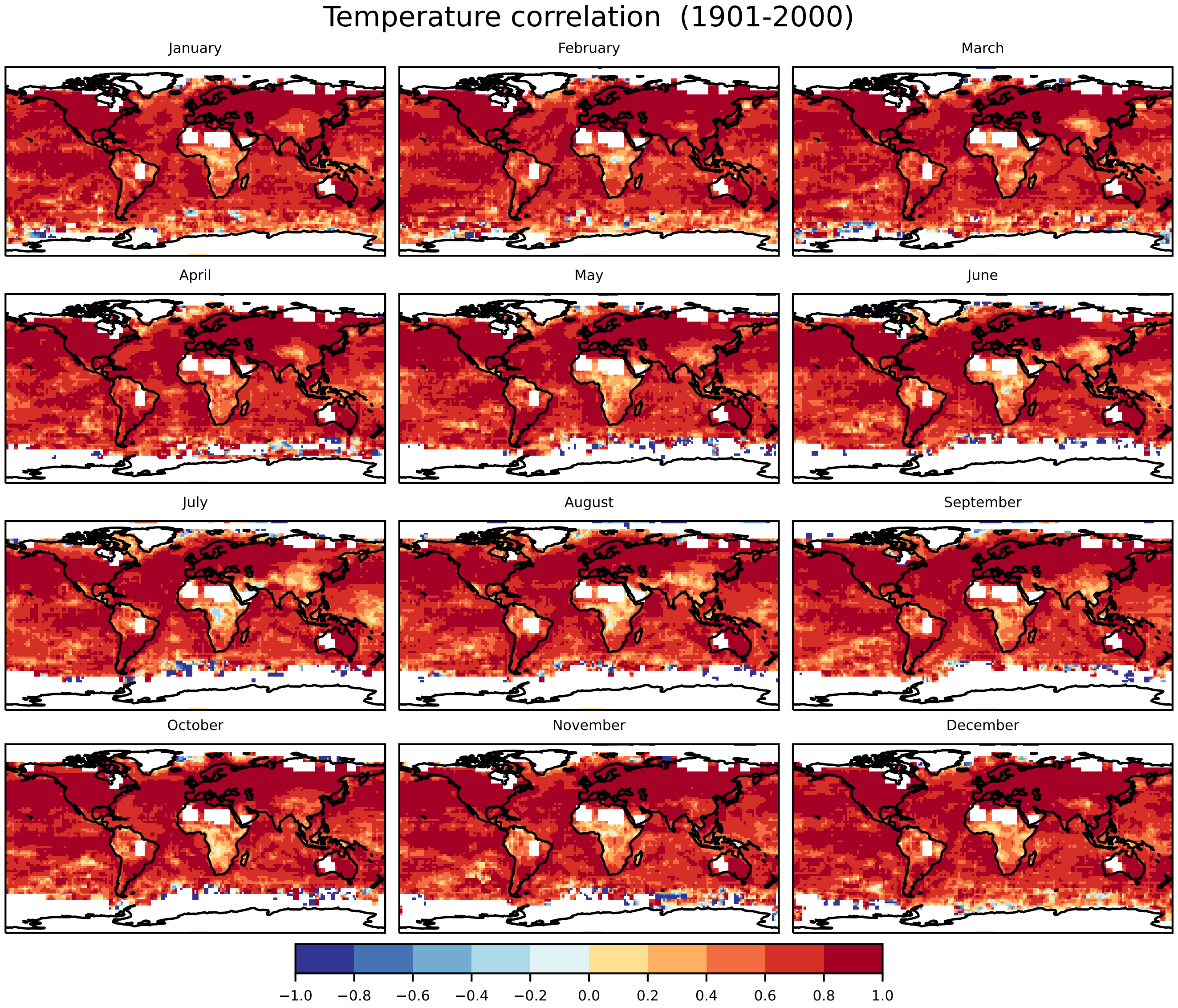

Figure S1. Correlation of monthly mean temperature calculated between the ensemble mean of ModE-RA and the HadCRUT5 reference dataset over the 1901-2000 time period.

ID	NAME	Location	Lon	Lat	Model	r_calib	r_ModE-RA
3	Norwich_plant_phenology_Snowdrop	Norwich	1.276	52.736	DecJan	0.353	0.374
4	Norwich_plant_phenology_Anemone	Norwich	1.275	52.736	NovDecJanFebMar	0.717	0.599
5	Norwich_plant_phenology_Hawthorn	Norwich	1.276	52.736	MarApr	0.638	0.659
6	Norwich_plant_phenology_Hawleaf	Norwich	1.276	52.736	OctNovDecJanFebMarApr	0.731	0.719
7	Norwich_plant_phenology_Sycmore	Norwich	1.276	52.736	FebMar	0.628	0.615
8	Norwich_plant_phenology_Birch	Norwich	1.276	52.736	OctNovFebMar	0.654	0.626
9	Norwich_plant_phenology_Elm	Norwich	1.276	52.736	Mar	0.485	0.517
10	Norwich_plant_phenology_Mountainash	Norwich	1.276	52.736	OctNovFebMarApr	0.807	0.795
12	Norwich_plant_phenology_Beech	Norwich	1.276	52.736	NovMarApr	0.695	0.637
13	Norwich_plant_phenology_Horsechestnut	Norwich	1.276	52.736	Mar	0.688	0.691
14	Norwich_plant_phenology_Chestnut	Norwich	1.276	52.736	DecJanFebMarApr	0.748	0.698
15	Norwich_plant_phenology_Hornbeam	Norwich	1.276	52.736	FebMar	0.502	0.570
16	Norwich_plant_phenology_Ash	Norwich	1.276	52.736	MarApr	0.602	0.616
17	Norwich_plant_phenology_Lime	Norwich	1.276	52.736	OctNovFebMarApr	0.782	0.712
18	Norwich_plant_phenology_Maple	Norwich	1.276	52.736	OctMarApr	0.532	0.595
101	YangtzeRiver	China	117.500	29.000	DecJanFeb	0.153	0.327
201	Amur_Blagoveshchensk_ice_phenology	Blagoveshchensk	127.633	50.250	Apr	0.743	0.751
202	Amur_Blagoveshchensk_ice_phenology	Blagoveshchensk	127.633	50.250	Jul	0.308	0.239
205	Angara_Bratsk_ice_phenology	Bratsk	101.833	56.067	DecMarAprMay	0.634	0.633
206	Angara_Bratsk_ice_phenology	Bratsk	101.833	56.067	JulAugNovDec	0.578	0.403

ID	NAME	Location	Lon	Lat	Model	r_calib	r_ModE-RA
211	Bullupe_Bolderaja_ice_phenology	Bolderaja	24.050	57.033	MarApr	0.806	0.803
212	Bullupe_Bolderaja_ice_phenology	Bolderaja	24.050	57.033	AugSep	0.420	0.442
229	Irtysh_Omsk_ice_phenology	Omsk	73.417	54.983	Apr	0.604	0.535
230	Irtysh_Omsk_ice_phenology	Omsk	73.417	54.983	JunJulAug	0.341	0.350
231	Iset_Yekaterinburg_ice_phenology	Yekaterinburg	60.617	56.833	Apr	0.713	0.738
235	Kama_Sarapul_ice_phenology	Sarapul	53.817	56.467	MarApr	0.801	0.792
237	Kama_Usolye_ice_phenology	Usolye	56.550	59.433	FebMarApr	0.768	0.808
239	Kama_Yelabuga_ice_phenology	Yelabuga	52.067	55.750	MarApr	0.736	0.763
240	Kama_Yelabuga_ice_phenology	Yelabuga	52.067	55.750	Jul	0.419	0.247
253	Lusu_Palema_ice_phenology	Palema	46.883	60.617	Apr	0.425	0.375
257	Narva_Narva_ice_phenology	Narva	28.200	59.383	MarApr	0.782	0.759
258	Narva_Narva_ice_phenology	Narva	28.200	59.383	JulAugSep	0.450	0.499
265	Ob_Salekhard_ice_phenology	Salekhard	66.600	66.517	May	0.513	0.471
267	Ob_Surgut_ice_phenology	Surgut	73.300	61.233	MarAprMay	0.786	0.793
268	Ob_Surgut_ice_phenology	Surgut	73.300	61.233	JunSep	0.549	0.460
269	Oka_Kagula_ice_phenology	Kagula	36.250	54.500	Mar	0.568	0.607
270	Oka_Kagula_ice_phenology	Kagula	36.250	54.500	Nov	0.299	0.286
277	Om_Omsk_ice_phenology	Omsk	73.417	54.983	FebMarApr	0.563	0.522
278	Om_Omsk_ice_phenology	Omsk	73.417	54.983	JunNov	0.288	0.240
283	Onega_Petrozadodsk_ice_phenology	Petrozadodsk	34.400	61.783	DecApr	0.678	0.656
285	Onega_Posad_ice_phenology	Posad	39.250	63.117	Apr	0.459	0.489
286	Onega_Posad_ice_phenology	Posad	39.250	63.117	Sep	0.349	0.395
293	Selenge_Novoselenginsk_ice_phenology	Novoselenginsk	106.900	51.100	Apr	0.407	0.335
295	Shchara_Slonim_ice_phenology	Slonim	25.333	53.100	NovFebMar	0.794	0.768
296	Shchara_Slonim_ice_phenology	Slonim	25.333	53.100	Oct	0.334	0.277
299	SyrDalya_Kazaly_ice_phenology	Kazaly	62.033	45.767	FebMar	0.624	0.670
303	Teza_Shuya_ice_phenology	Shuya	41.400	56.850	JanFebMar	0.536	0.566
305	Tom_Tomsk_ice_phenology	Tomsk	84.967	56.500	MarAprMay	0.761	0.711
312	Ural_Orenburg_ice_phenology	Orenburg	55.117	51.750	Sep	0.436	0.426
317	Vaga_Verkhovasje_ice_phenology	Verkhovasje	42.050	60.750	Apr	0.719	0.709
327	Volga_Kazan_ice_phenology	Kazan	49.117	55.783	MarApr	0.599	0.610
328	Volga_Kazan_ice_phenology	Kazan	49.117	55.783	Jul	0.387	0.372
329	Volga_NizhnyNovgorod1_ice_phenology	NizhnyNovgorod1	43.617	56.500	DecJanFebMar	0.538	0.545
330	Volga_NizhnyNovgorod1_ice_phenology	NizhnyNovgorod1	43.617	56.500	Jul	0.445	0.473
333	Volga_Saratov_ice_phenology	Saratov	46.050	51.533	JanFebMar	0.467	0.417
334	Volga_Saratov_ice_phenology	Saratov	46.050	51.533	Aug	0.311	0.148
337	Volga_Ulyanovsk_ice_phenology	Ulyanovsk	48.417	54.317	JanFebMarApr	0.723	0.698
339	Volga_Volgograd_ice_phenology	Volgograd	44.517	48.700	DecJanFebMar	0.710	0.669
340	Volga_Volgograd_ice_phenology	Volgograd	44.517	48.700	Sep	0.491	0.490
341	Volga_Yuryevets_ice_phenology	Yuryevets	43.117	57.317	NovMarApr	0.812	0.769
355	Vytegra_Vytegra_ice_phenology	Vytegra	36.450	61.000	Apr	0.700	0.649
357	Yenisey_Krasnojarsk_ice_phenology	Krasnojarsk	92.867	56.017	MarAprMay	0.683	0.551
358	Yenisey_Krasnojarsk_ice_phenology	Krasnojarsk	92.867	56.017	Aug	0.181	0.071
1102	Eastriver_Eastmain_ice_phenology	Eastmain	−78.518	52.234	AprMay	0.674	0.669
1104	Nunatsiavut_Nunatsiavut_ice_phenology	Nunatsiavut	−66.500	56.500	Sep	0.406	0.211
1105	Nunatsiavut_Nunatsiavut_ice_phenology	Nunatsiavut	−66.500	56.500	Apr	0.451	0.328
1213	Vyatka_River_Slobodskoy_ice_phenology	Slobodskoy	50.200	58.733	MarApr	0.487	0.458
1221	Lieksanjoki_Lieksa_ice_phenology	Lieksa	30.067	63.317	Apr	0.417	0.410
1451	HudsonBayWest_Sea_Ice	Churchill_YorkFactory	−93.200	58.200	Aug	0.271	0.113
1452	HudsonBayEast_Sea_Ice	MooseFactory	−80.593	51.263	AprMayJun	0.441	0.145
1455	HudsonStraight_HudsonStraight	HudsonStraight	−70.000	62.500	Apr	0.269	0.065

Table S1. Correlation between independent documentary series from DocuClim and calibration series as well as with the ensemble mean of ModE-RA.

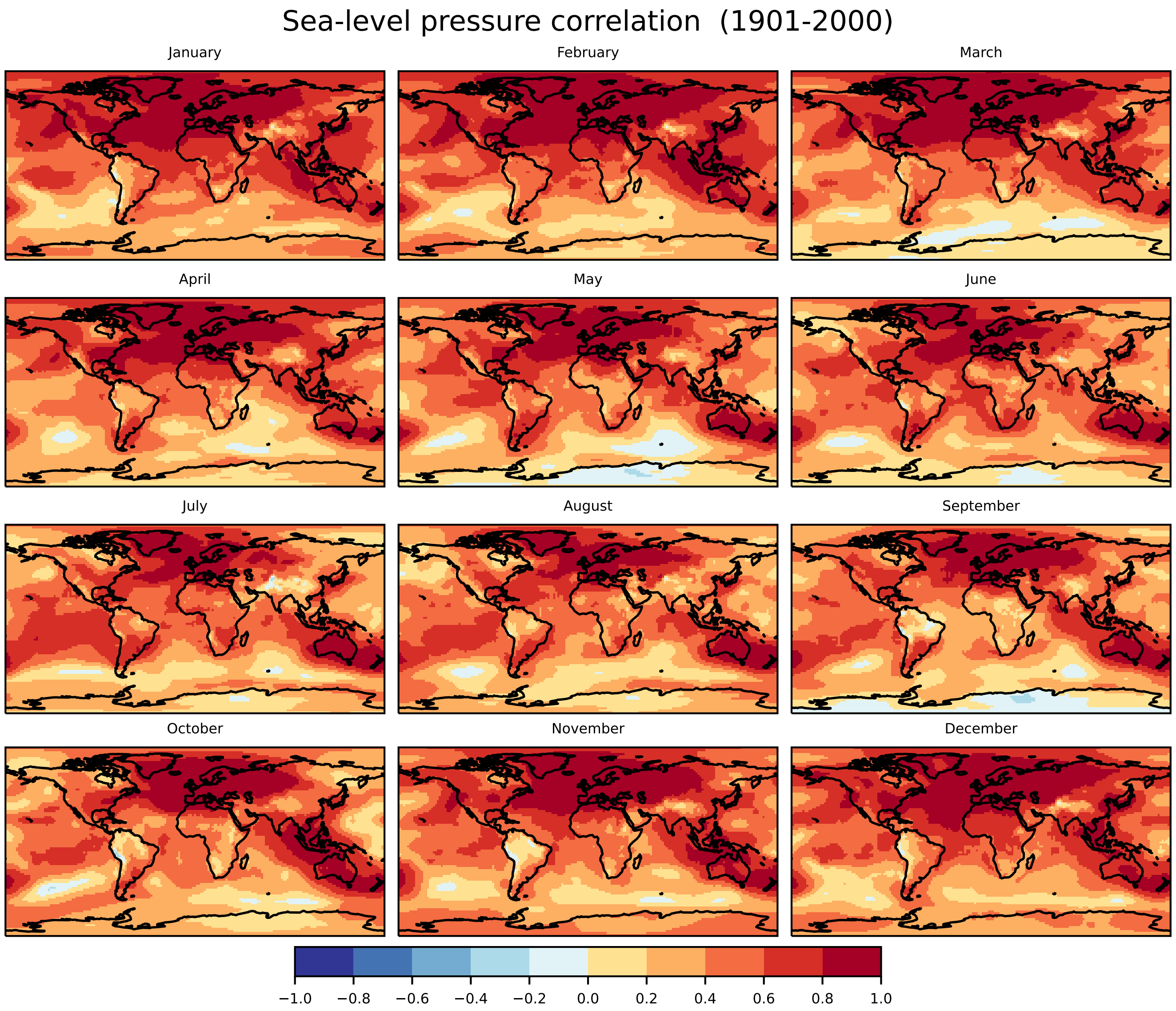

Figure S2. Correlation of monthly mean sea-level pressure calculated between the ensemble mean of ModE-RA and the HadSLP2 reference dataset over the 1901-2000 time period.

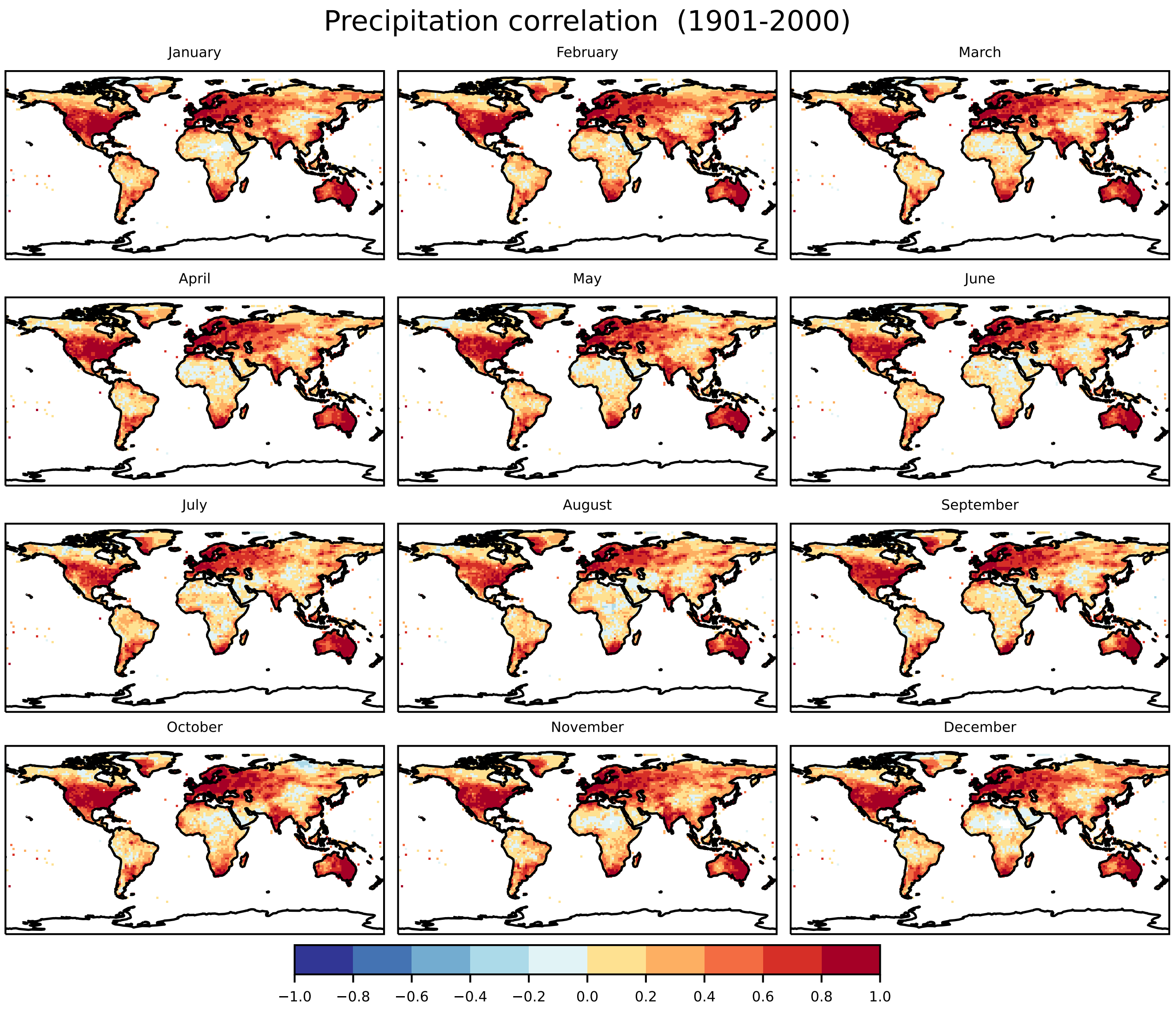

Figure S3. Correlation of monthly precipitation sums calculated between the ensemble mean of ModE-RA and the GPCC reference dataset over the 1901-2000 time period.

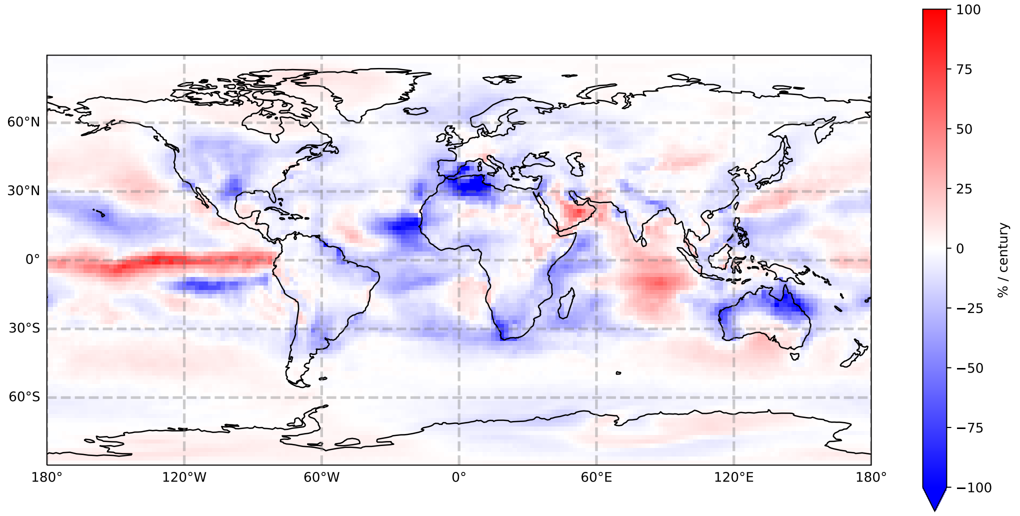

Figure S4. Linear trend of annual precipitation sums divided by the 19th century precipitation climatology in the ModE-RA ensemble mean. I.e. a value of 10 indicates a 10% increase over the 19th century.)
